# Supplementary material for: Impaired Cardiac Sympathetic Innervation Increases the Risk of Cardiac Events in Heart Failure Patients with Left Ventricular Hypertrophy and Mechanical Dyssynchrony
Source: J Clin Med. 2021 Oct 28;10(21):5047. doi: 10.3390/jcm10215047 (PMC8584654; doi:10.3390/jcm10215047)
Supplement: Supplementary file 1 [file jcm-10-05047-s001.zip › jcm-1402420-supplementary.pdf]

Figure S1.

Phase SD and LVMI, standardized late HMR in three dimensions cut-off value determined by ROC analysis for the prediction of cardiac events

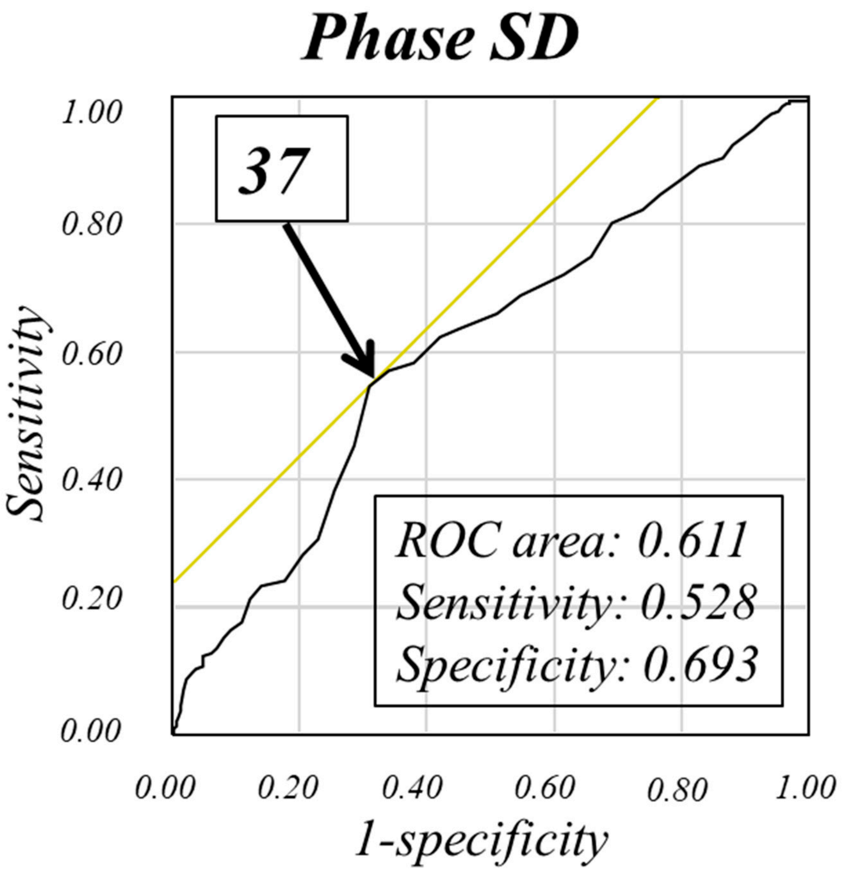

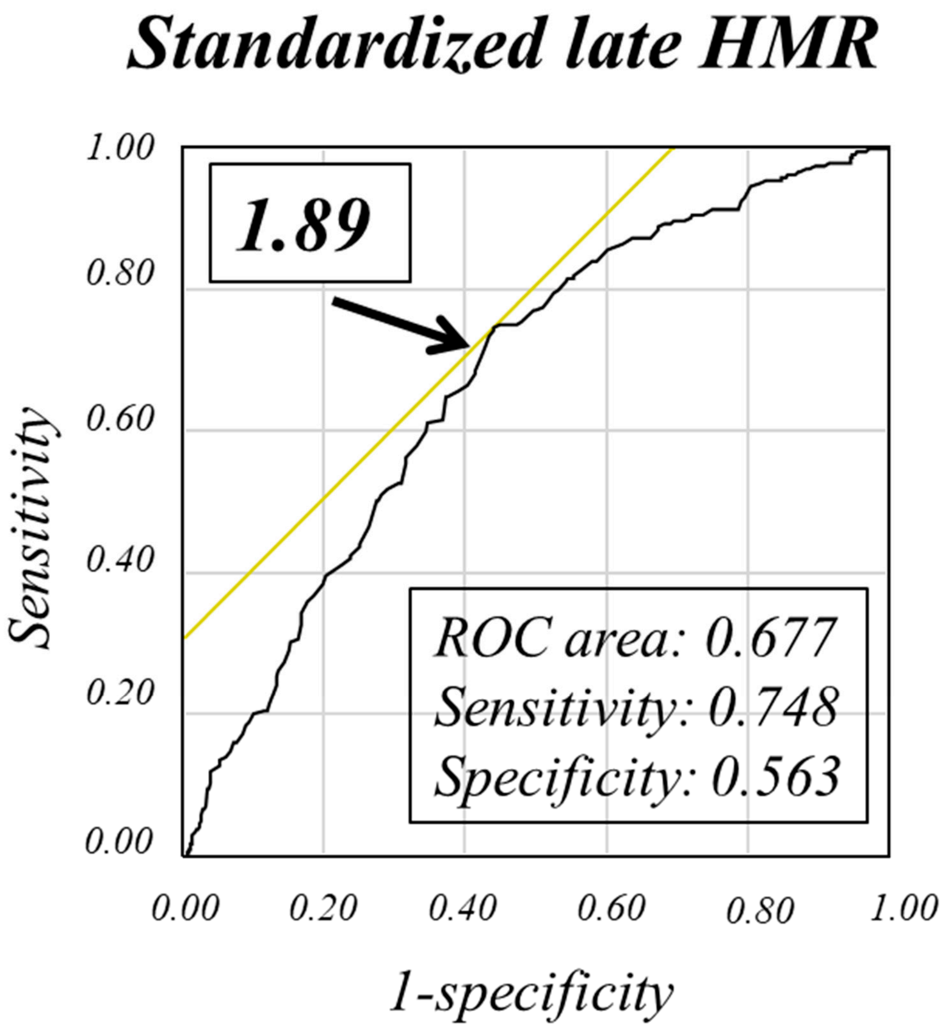
Standardized late HMR
1.89
ROC area: 0.677
Sensitivity: 0.748
Specificity: 0.563
Sensitivity
1-specificity
0.00
0.20
0.40
0.60
0.80
1.00

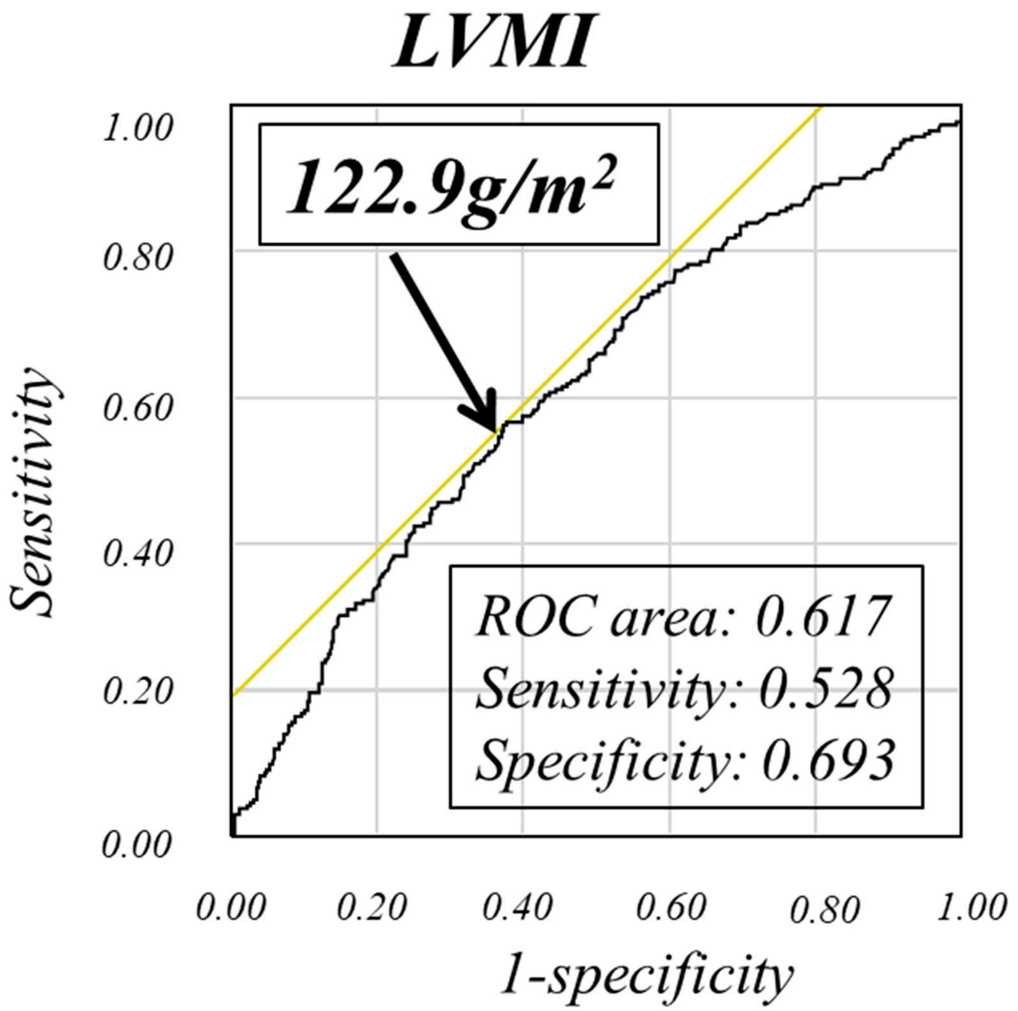
LVMI
122.9g/m2
ROC area: 0.617
Sensitivity: 0.528
Specificity: 0.693
Sensitivity
1-specificity
0.00
0.20
0.40
0.60
0.80
1.00

Figure S2.

(A). Phase SD and LVMI in three dimensions cut-off value determined by ROC analysis for the prediction of cardiac events combination with three parameters in the ischemic etiology group. (B). Phase SD and LVMI in three dimensions cut-off value determined by ROC analysis for the prediction of cardiac events combination with three parameters in the non-ischemic etiology group.

(A) **(B)**

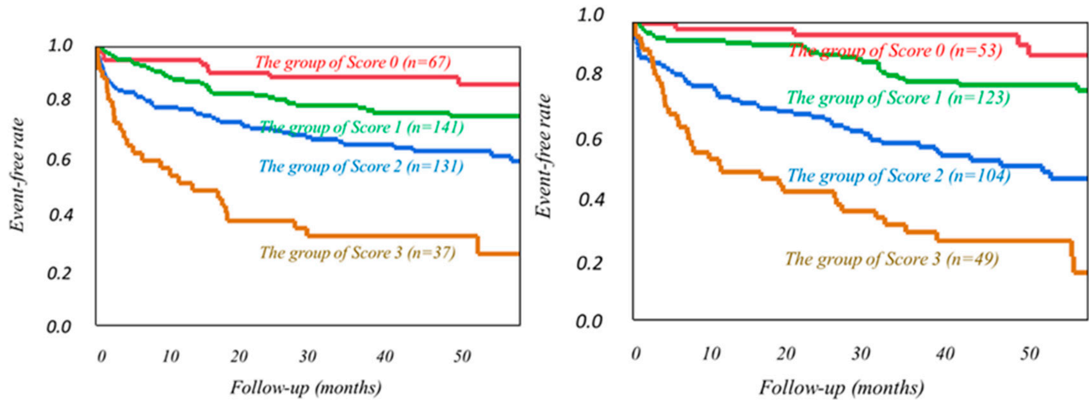

Group3 vs Group0 Log-Rank 56.0,	P < 0.0001
Group3 vs Group1 Log-Rank 54.1,	P < 0.0001
Group3 vs Group2 Log-Rank 9.95,	P = 0.0016
Group1 vs Group2 Log-Rank 23.5,	P < 0.0001
Group1 vs Group0 Log-Rank 6.59,	P = 0.0102
Group2 vs Group0 Log-Rank 29.8,	P < 0.0001

Group3 vs Group0 Log-Rank 44.4,	P < 0.0001
Group3 vs Group1 Log-Rank 41.7,	P < 0.0001
Group3 vs Group2 Log-Rank 14.3,	P = 0.0001
Group1 vs Group2 Log-Rank 7.24,	P = 0.0071
Group1 vs Group0 Log-Rank 3.99,	P = 0.0456
Group2 vs Group0 Log-Rank 14.5,	P = 0.0001

Scoring

Phase SD>37;1 Phase SD≦37;0

Standardized late HMR≧1.89;0 Standardized late HMR<1.89;1

LVMI>122.9;1 LVMI≦122.9;0

The total number of scores added together was used as the group number of the registered patients.

(C). Phase SD and LVMI in three dimensions cut-off value determined by ROC analysis for the prediction of cardiac events combination with three parameters in the group of males. (D). Phase SD and LVMI in three dimensions cut-off value determined by ROC analysis for the prediction of cardiac events combination with three parameters in the group of females.

(C) **(D)**

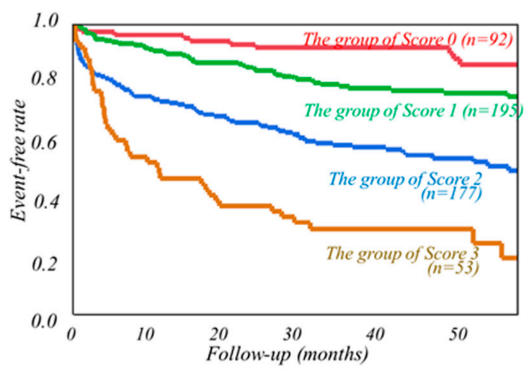

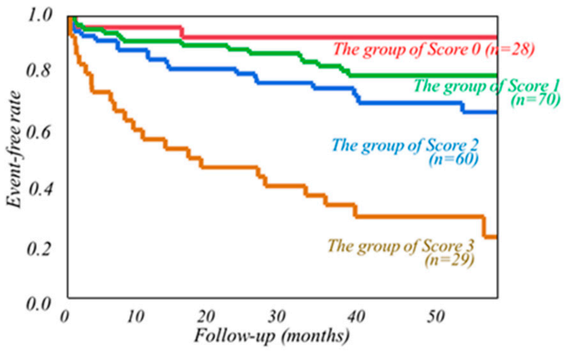

Group3 vs Group0 Log-Rank 76.4,	P < 0.0001
Group3 vs Group1 Log-Rank 71.2,	P < 0.0001
Group3 vs Group2 Log-Rank 14.1,	P < 0.0001
Group1 vs Group2 Log-Rank 25.1,	P < 0.0001
Group1 vs Group0 Log-Rank 8.24,	P = 0.0041
Group2 vs Group0 Log-Rank 38.1,	P < 0.0001

Group3 vs Group0 Log-Rank 25.6,	P < 0.0001
Group3 vs Group1 Log-Rank 32.5,	P < 0.0001
Group3 vs Group2 Log-Rank 16.3,	P < 0.0001
Group1 vs Group2 Log-Rank 4.01,	P = 0.0432
Group1 vs Group0 Log-Rank 3.96,	P = 0.0450
Group2 vs Group0 Log-Rank 6.17,	P = 0.0130

Scoring
Phase SD>37;1 Phase SD≦37;0
Standardized late HMR≧1.89;0 Standardized late HMR<1.89;1
LVMI>122.9;1 LVMI≦122.9;0
The total number of scores added together was used as the group number of the registered patients.

(E). Phase SD and LVMI in three dimensions cut-off value determined by ROC analysis for the prediction of cardiac events combination with three parameters in the group < 65 years old. (F). Phase SD and LVMI in three dimensions cut-off value determined by ROC analysis for the prediction of cardiac events combination with three parameters in the group ≧65 years old.

(E)

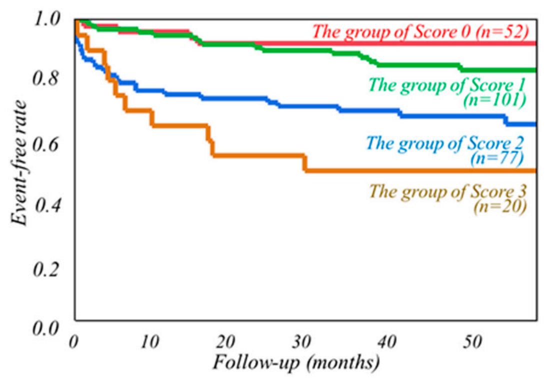

Group3 vs Group0 Log-Rank 18.2,	P < 0.0001
Group3 vs Group1 Log-Rank 17.3,	P < 0.0001
Group3 vs Group2 Log-Rank 14.1,	P = 0.0001
Group1 vs Group2 Log-Rank 13.9,	P = 0.0002
Group1 vs Group0 Log-Rank 0.92,	P = 0.3387
Group2 vs Group0 Log-Rank 14.9,	P = 0.0001

(F)

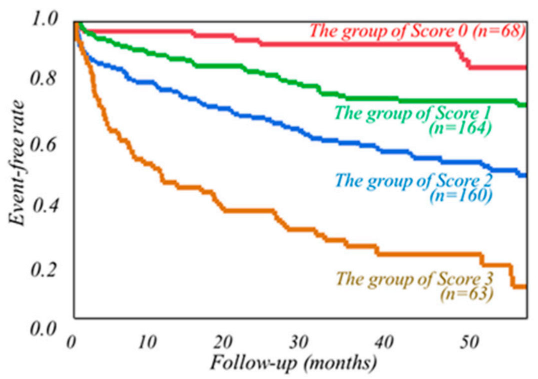

Group3 vs Group0 Log-Rank 75.0,	P < 0.0001
Group3 vs Group1 Log-Rank 74.9,	P < 0.0001
Group3 vs Group2 Log-Rank 27.6,	P < 0.0001
Group1 vs Group2 Log-Rank 12.7,	P = 0.0004
Group1 vs Group0 Log-Rank 9.89,	P = 0.0017
Group2 vs Group0 Log-Rank 27.6,	P < 0.0001

Scoring
Phase SD>37;1 Phase SD≦37;0
Standardized late HMR≧1.89;0 Standardized late HMR<1.89;1
LVMI>122.9;1 LVMI≦122.9;0
The total number of scores added together was used as the group number of the registered patients.

(G). Phase SD and LVMI in three dimensions cut-off value determined by ROC analysis for the prediction of cardiac events combination with three parameters in the group with EF < 40%. (H). Phase SD and LVMI in three dimensions cut-off value determined by ROC analysis for the prediction of cardiac events combination with three parameters in the group with EF≧40%.

(G) **(H)**

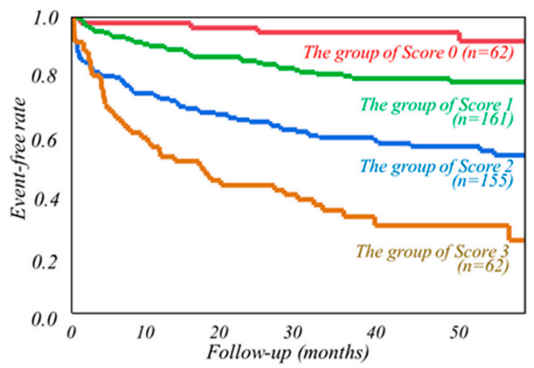

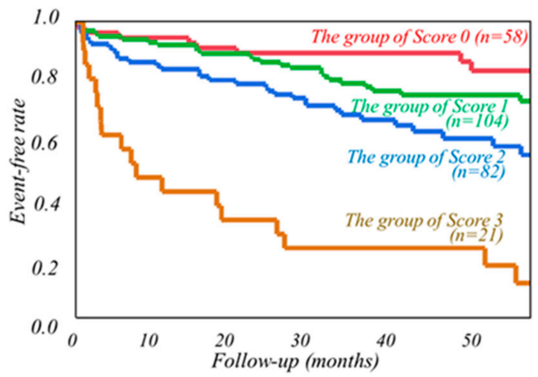

Group3 vs Group1 Log-Rank 59.1,	P < 0.0001
Group3 vs Group2 Log-Rank 10.8,	P < 0.0001
Group3 vs Group0 Log-Rank 57.1,	P < 0.0001
Group1 vs Group2 Log-Rank 22.1,	P < 0.0001
Group1 vs Group0 Log-Rank 8.19,	P = 0.0042
Group2 vs Group0 Log-Rank 79.5,	P < 0.0001

Group3 vs Group0 Log-Rank 45.2,	P < 0.0001
Group3 vs Group1 Log-Rank 45.0,	P < 0.0001
Group3 vs Group2 Log-Rank 18.3,	P < 0.0001
Group1 vs Group2 Log-Rank 5.95,	P = 0.0147
Group1 vs Group0 Log-Rank 2.88,	P = 0.0895
Group2 vs Group0 Log-Rank 13.2,	P = 0.0003

Scoring
Phase SD>37;1 Phase SD≦37;0
Standardized late HMR≧1.89;0 Standardized late HMR<1.89;1
LVMI>122.9;1 LVMI≦122.9;0
The total number of scores added together was used as the group number of the registered patients.

Table S1. CONSORT 2010 checklist of information to include when reporting a randomised trial*

Section/Topic	Item No	Checklist item	Reported on page No
		**Title and abstract**	
	1a	Identification as a randomised trial in the title	Page1 line.1
	1b	Structured summary of trial design, methods, results, and conclusions (for specific guidance see CONSORT for abstracts)	Page2 line.1-19
		**Introduction**	
Background and objectives	2a	Scientific background and explanation of rationale	Page3 line.1-Page4 line.14
	2b	Specific objectives or hypotheses	Page4 line15-18
		**Methods**	
Trial design	3a	Description of trial design (such as parallel, factorial) including allocation ratio	Page4 line.22-23
	3b	Important changes to methods after trial commencement (such as eligibility criteria), with reasons	Nothing
Participants	4a	Eligibility criteria for participants	Page5 lin.1-10
	4b	Settings and locations where the data were collected	Page4 line22-23
Interventions	5	The interventions for each group with sufficient details to allow replication, including how and when they were actually administered	This was an observation al study, so there was no intervention.
Outcomes	6a	Completely defined pre-specified primary and secondary outcome measures, including how and when they were assessed	Page7 line.21-Page8 line.2.
	6b	Any changes to trial outcomes after the trial commenced, with reasons	
Sample size	7a	How sample size was determined	Page4 line.22-23
	7b	When applicable, explanation of any interim analyses and stopping guidelines	Since this was an observation al study, there were no stopping criteria.
Randomisation:			
Sequence generation	8a	Method used to generate the random allocation sequence	Since this was an observation al study, there was

			no random assignment.
	8b	Type of randomisation; details of any restriction (such as blocking and block size)	Since this was an observational study, there was no random assignment.
Allocation concealment mechanism	9	Mechanism used to implement the random allocation sequence (such as sequentially numbered containers), describing any steps taken to conceal the sequence until interventions were assigned	Mechanism used to implement the random allocation sequence (such as sequentially numbered containers), describing any steps taken to conceal the sequence until interventions were assigned
Implementation	10	Who generated the random allocation sequence, who enrolled participants, and who assigned participants to interventions	Since this was an observational study, there was no intervention and no random assignment.
Blinding	11a	If done, who was blinded after assignment to interventions (for example, participants, care providers, those assessing outcomes) and how	This was an observational study, so there was no randomization after the intervention.
	11b	If relevant, description of the similarity of interventions	This was an observational study, so there was no intervention.
Statistical methods	12a	Statistical methods used to compare groups for primary and secondary outcomes	Page8 line.7-20.
	12b	Methods for additional analyses, such as subgroup analyses and adjusted analyses	No sub-analysis was performed in this study.

Results

Participant flow (a diagram is strongly recommended)	13a	For each group, the numbers of participants who were randomly assigned, received intended treatment, and were analysed for the primary outcome	Page8 line.22-Page9 line.5
	13b	For each group, losses and exclusions after randomisation, together with reasons	This was an observational study, so there were no patient dropouts or missing data.
Recruitment	14a	Dates defining the periods of recruitment and follow-up	This was an observational study, so there were no patient dropouts or missing data.
	14b	Why the trial ended or was stopped	The present study was a no-intervention study within the observation period.
Baseline data	15	A table showing baseline demographic and clinical characteristics for each group	Page8 line.22-Page9 line.5
Numbers analysed	16	For each group, number of participants (denominator) included in each analysis and whether the analysis was by original assigned groups	In this study, we divided the results into the cardiac event group and the non-cardiac event group.
Outcomes and estimation	17a	For each primary and secondary outcome, results for each group, and the estimated effect size and its precision (such as 95% confidence interval)	It was shown in table3.
	17b	For binary outcomes, presentation of both absolute and relative effect sizes is recommended	This time, only the cardiac accident was used as the primary outcome.
Ancillary analyses	18	Results of any other analyses performed, including subgroup analyses and adjusted analyses, distinguishing pre-specified from exploratory	No subgroup analysis or adjusted analysis was performed

			in this study.
Harms	19	All important harms or unintended effects in each group (for specific guidance see CONSORT for harms)	There were no adverse events during the observation period.
		**Discussion**	
Limitations	20	Trial limitations, addressing sources of potential bias, imprecision, and, if relevant, multiplicity of analyses	Page32 line. 3 - Page33 line.12
Generalisability	21	Generalisability (external validity, applicability) of the trial findings	Page29 line.8- Page32 line.2
Interpretation	22	Interpretation consistent with results, balancing benefits and harms, and considering other relevant evidence	Page33 line14-16
		**Other information**	
Registration	23	Registration number and name of trial registry	2016-074
Protocol	24	Where the full trial protocol can be accessed, if available	Since this was an observation al study, there was no special protocol.
Funding	25	Sources of funding and other support (such as supply of drugs), role of funders	No funding

*We strongly recommend reading this statement in conjunction with the CONSORT 2010 Explanation and Elaboration for important clarifications on all the items. If relevant, we also recommend reading CONSORT extensions for cluster randomised trials, non-inferiority and equivalence trials, non-pharmacological treatments, herbal interventions, and pragmatic trials. Additional extensions are forthcoming: for those and for up to date references relevant to this checklist, see www.consort-statement.org.
